# Do you really believe that? The effect of economic incentives on the acceptance of real-world data in a polarized context

**DOI:** 10.1098/rsos.240252

**Published:** 2024-04-24

**Authors:** Mike Farjam, Giangiacomo Bravo

**Affiliations:** ^1^ Institute for Journalism and Communication Research, Universität Hamburg, Hamburg, Germany; ^2^ Department of Social Studies and Centre for Data Intensive Sciences and Applications, Linnaeus University, Växjö, Sweden

**Keywords:** motivated reasoning, false polarization, perceived polarization, attitude–behaviour gap, incentives

## Abstract

Attitudes and expectations towards others are major drivers of political polarization. However, there is limited understanding of their relevance when decisions with high stakes are taken. In this study, we compare self-reported attitudes against economically incentivized estimates of data coming from official sources and offer participants financial rewards for accuracy. Our methodology yields three principal findings. (i) Extreme attitudes from a small partisan subgroup primarily account for the observed partisan divide; this subgroup diminishes when incentivized estimates are considered. (ii) There is a weak correlation between economically incentivized and unincentivized measures within individual respondents. (iii) We introduce a novel metric for assessing perceived polarization. This metric allows participants to estimate data points for those with opposing political views, rewarding accurate predictions financially. Interestingly, this measure of perceived polarization correlates with attitudes but not with incentivized data estimates. This is in line with the concept of ‘false polarization’, attributing polarization more to expectations towards others than to genuine differences. These findings challenge the reliability of standard attitude surveys and suggest avenues for mitigating perceived polarization in contentious issues.

## 1. Introduction

Emerging and enduring challenges, from climate change to the COVID-19 pandemic, reveal how conflicts between political interests and scientific evidence complicate rational discourse and thwart effective policy solutions [1–3]. This intermingling often leads to motivated reasoning, where mechanisms of partisan identity defence activate, effectively isolating individuals from opposing viewpoints and heightening the polarization of the debate [4].

At the same time, evidence suggests that polarization within the political discourse might be superficial. When discussions pivot from abstract norms to tangible policy options, the apparent polarization often diminishes [5,6]. Research also indicates that public declarations of values and norms often serve to bolster in-group status, even when privately held views diverge [7,8]. Thus, social sorting based on political affiliation, rather than ideological differences, could be the principal catalyst for increasing polarization [9]. Moreover, a pronounced attitude–behaviour gap exists in domains, such as environmental and health issues, suggesting that publicly expressed opinions may not accurately reflect private actions [10–12]. These considerations raise the question of whether people who express extreme and factually false beliefs on a politically polarized issue—for instance, that climate change is not occurring or has no anthropic causes—really believe the statements that they are expressing and would act accordingly when real stakes are at play—for instance, refusing to insure their houses against an increased risk of flooding.

A significant stream of the literature also shows that incorrect perceptions about the positions and values of members of the opponent group can greatly increase perceived polarization and intergroup hostility [13,14]. This ‘false polarization’ is dangerous because it exacerbates the perceived differences between partisan groups and hinders compromise [6]. Similar to actual polarization, does false polarization actually play a role once high stakes are at play or is false polarization primarily a concern when dealing with others during low-stakes situations?

To address these questions, we take a series of controlled measurements and compare self-reported attitudes on contentious political issues against economically incentivized estimates of factual data on those issues. Participants received financial rewards based on the accuracy of their estimates of the data, introducing tangible stakes into their responses. Our study design is grounded in economic theories of revealed preferences, which argue that incentivized actions reveal genuine beliefs more reliably than non-incentivized responses, often considered mere ‘cheap talk’ [15,16].

In comparing responses to politicized factual questions with and without incentives for correctness, we followed a conceptual design introduced by Bullock *et al.* [17], who showed that small payments for correct and ‘don’t know’ answers sharply diminished the gap between partisan groups. Prior *et al.* [18] similarly found that partisan differences in reports of economic conditions were reduced when the participants were motivated through incentives to give correct answers. Interestingly, Robbett & Matthews [19] found that Republicans and Democrats were less likely to provide different answers to factual questions when asked in an individual setting compared with anonymously voting in a group on that issue. The authors interpret this as evidence for *expressive voting*, that is, that individuals do not just express their own views on an issue when voting; votes are also an expression of and conforming to partisan views [20].

Our study contributes to this literature with three key innovations. First, we ask participants similar questions, once in terms of agreeance with politically polarized statements and once by making them estimate objective data underlying these statements. For the second type of questions, we provide financial incentives for correctness. We, thereby, identify characteristics of individuals associated with (in)consistencies between both types of questions. Second, while previous studies asked agree/disagree questions, we ask participants to make point estimates, thereby allowing us to observe different degrees and directions of partisan bias. Third, by making participants guess estimates of individuals from the opposing partisan group and again incentivizing them for correctness, we provide, to the best of our knowledge, a first incentivized measure of perceived polarization [21].

We asked people living in the United States regarding their views and beliefs on two contentious issues that were chosen to be (i) highly politically debated within the United States and (ii) with the actual reality of the data typically contested across parties. The issues were *Global warming*—here preferred to the more correct expression ‘climate change’, because it is at the core of the current political debate [22]—where beliefs expressed by Democrats tend to be more aligned with empirical data [23], and the relationship between the racial composition of an area and homicide rates (henceforth, *Race and crime*), where Republicans’ beliefs are possibly closer to data [24]. We expect unincentivized attitudes to differ along partisan lines in survey-like measures but differences to attenuate with the introduction of incentives and when referring to actual data. This leads to the following hypotheses:

**Hypothesis 1 (H1):** Self-reported support for statements on global warming is lower for Republicans than Democrats, while the opposite is observed when statements concern the link between race and crime.
**Hypothesis 2 (H2):** Incentivized estimates from both Republicans and Democrats concerning historical temperature variations and the relationship between racial demographics and crime rates will show no significant differences.
**Hypothesis 3 (H3):** Both groups will anticipate that the opposing party’s incentivized estimates will diverge from their own, reflecting the publicly stated attitudes rather than the incentivized estimates.

## 2. Methods

In a nutshell, our study works as follows: we randomly assigned participants to either the *Global warming* or the *Race and crime* questionnaire to ensure that demographics and partisanship were similarly distributed. All participants answered the following three questions in a fixed order. First, they indicated their general attitudes on the issue, then they estimated data referring to the issue, and finally, they guessed by how much their own data estimate differed from opposing partisans. We want to test to what extent the three questions are answered along partisan lines. Table 1 provides the exact wording of the three questions in both questionnaires.

**Table 1.** Overview of issues and measures in the questionnaire.

measure	issue	
	*global warming*	*race and crime*
*attitude* (non-incentivized question)	temperatures in the United States have increased drastically during the last 100 years (indicate agreement)	generally, the more African or Black American people live in an area in the United States, the higher the crime rate in the area (indicate agreement)
*estimate* (incentivized task)	estimate the temperature difference between the 1950s and 2010s in a random US state	estimate the difference in homicide mortality between the US states with the third highest and third lowest African/Black Americans’ proportion
*estimate difference* (incentivized task)	difference of estimate between (myself) and someone with opposing partisan preferences	difference of estimate between (myself) and someone with opposing partisan preferences

Each question was designed to measure an outcome variable corresponding to our three research hypotheses. In stage 1, similar to a typical survey, we measured attitudes within a politically polarized issue and general beliefs about the levels of polarization in the United States. A pivotal aspect of our study is the incentivization of guesses during stages 2 and 3. Specifically, participants were awarded a substantial bonus—up to $2—if their estimates closely matched the actual data. We contrast incentivized and unincentivized measures to identify the potential gaps between expressed attitudes and actual beliefs of participants.

Stage 1 adopted a conventional survey format, the details of which can be found in the electronic supplementary material. Participants were asked a series of socio-demographic questions (used as controls in our regression models) along with questions on their political views. A crucial element in our analysis involved querying participants about their partisan affiliations. Owing to stage 3, which required pairing participants with others holding opposite partisan views, we operationalized this measure on a binary Republican/Democrat scale. Additionally, we assessed participants’ perceptions of national polarization using a 0−10 scale. This was done both generally and in relation to the issue under study—either ‘Global Warming’ or ‘Race and Crime’. Stage 1 ended with a series of five statements on the questionnaire issue, where participants had to indicate their level of agreement on a 0−10 scale.

Among these five statements, one was especially relevant for our study. In the Global warming questionnaire, this was *Temperatures in the United States have increased drastically during the last 100 years*. In the Race and crime questionnaire, it was *Generally, the more African or Black American people live in an area in the United States, the higher the crime rate in that area*. Despite the fact that statistics from reputable government agencies provide strong evidence that both statements are valid at an aggregate level (see data sources used below), we expect that owing to the politically polarized context in which they are embedded, participants would answer these questions along typical partisan lines. In other words, in the absence of clear incentives to more carefully reflect on the statement or to express conviction in line with real-world data, we expect participants to react to these items by reporting what represents the standard answer in their social and political environments [25]. This level of agreement with the pertinent statement constitutes the principal metric for stage 1 and will hereafter be denoted as the participants’ *attitude*.

Note that the statements through which attitude was measured were carefully selected to not include any claims about the causes behind the observed data. That is, the statements did not claim that warming was anthropogenic or that race was causing crime rates to differ. Consequently, variances in attitude across partisan groups should not stem from divergent beliefs about the root causes underlying the observed data. Furthermore, we chose to embed the attitude item together with other related attitude items (with statements that are harder to verify or for which an objective truth may not exist) for several reasons. First, multiple attitude items are usually bundled in surveys to create an aggregated index. Thus, our study serves as an examination of the external validity of such attitude items, specifically examining their applicability in high-stakes contexts. We acknowledge that agreement with a single statement represents a less precise measure of attitudes than the batteries of items commonly used in questionnaires. Nevertheless, such individual items correlate with the latent variable of interests and, thereby, our measure stands as a proxy for the typical ways in which attitudes tend to be measured. Second, by bundling an item with others, we are embedding a factual question (e.g. is there a statistical relationship between crime and race) with other less factual statements (e.g. race relations in the United States are bad). This design emulates conventional surveys, which often conflate factual knowledge, evidence-based beliefs, and personal opinions.

In stage 2, instead of asking participants about their agreement with statements on the questionnaire issues, we asked them to estimate the value of actual data. In the Global warming questionnaire, participants had to estimate the recorded difference in average temperatures between the 1950s and 2010s for a random US state, as reported by the National Centers for Environmental Information (NCEI). Participants were informed that a correct estimate would earn them a $2 bonus, with reductions of $0.1 for every 0.1°F deviation from the actual value. Errors exceeding 2°F would nullify the bonus entirely.

In the questionnaire on race and crime, participants were tasked with estimating the disparity in homicide mortality rates—measured in deaths per 1000 residents—between two US states for a randomly selected year. This difference was computed based on data from the Centers for Disease Control and Prevention (CDC). Instead of being told which state was selected, they were only informed that state X was the state with the third highest and state Y was the state with the third lowest percentage of black or African American population. Participants were required to gauge the multiplicative factor by which the homicide mortality rate in one state exceeded that in the other. Again, participants received a $2 bonus if their guess was correct and a reduction of $0.1 for each decimal point of error.

A potential critique on our questionnaire is that participants may not trust the official sources to report reliable data about the issue. To control for this potential confound, prior to initiating stage 2, participants were asked to express their level of trust in the data sources underpinning the correct answers (i.e. CDC and NCEI). They were then notified that the data used in the questionnaire were derived from these institutions. The level of trust was later used in our analysis as a control variable. Furthermore, since the data were publicly available, the participants were only given 60 s to enter their estimates to avoid searching for the correct answer online.

In stage 3, participants were informed that they had to guess another participant’s estimate from stage 2. The only information that they received was that the other participant had opposing party preferences and that they would earn an additional $2 if they correctly guessed the estimate that the other participant made during stage 2. This amount was reduced based on their guessing error as above. For our analysis, the difference between an individual participant’s own estimate (from stage 2) and their anticipated estimate of another participant (from stage 3) serves as our incentivized metric for assessing perceived polarization in the issue under investigation. This will be referred to as the *Incentivized Estimate Difference* henceforth.

As a note of methodological caution, we emphasize that we opted for a questionnaire design where all stages had a *pre-determined order*—that is, the stages were not administered in a randomized fashion. This design strategy was employed to facilitate the detection of internal inconsistencies among participants. The adopted design may predispose participants to generate incentivized estimates that align with their previously expressed attitudes. Given that such a consistency bias [26] would undermine the validity of Hypothesis 2 (H2), any support found for H2 can be interpreted as a conservative estimate of the discrepancy between stated attitudes and real data beliefs in real-world contexts. Subsequent research that does not prioritize such comparisons of several answers for the same participant may opt to randomly assign the order of measurements.

## 3. Results

### 3.1. Sample

The study was conducted in July 2022, using the Amazon Mechanical Turk (MTurk) crowdsourcing platform and oTree [27]. In accordance with our pre-registered analysis plan,^1^ a total of 902 participants provided informed consent and were subsequently incorporated into the final analysis. Participants took on average 7 min to complete the study and received a $1.5 show-up fee plus a bonus of up to $4 depending on the correctness of their estimates. Eligibility criteria mandated participants to be US residents—verified through IP address screening and an MTurk filter—and at least 18 years old. In our sample, 56% were male, and the average age was 35.7 (s.d. = 11.0). Previous empirical evidence suggests that MTurk samples exhibit demographic characteristics closely mirroring those of the US population, relative to conventional laboratory-based samples [28,29]. It should be noted, however, that Democrats are often overrepresented, as evidenced by a 71% representation in our sample. However, since we compare statistics between partisan groups in our hypotheses, this imbalance risks, if anything, to reduce the power of our statistical tests. The results presented in table 2 may hence be seen as a conservative test for our hypotheses. In addition, we control in our regression models for the most common socio-demographic variables. Electronic supplementary material, table S5, presents balance statistics for both issues.

**Table 2.** Regression models formally testing our hypotheses and including demographic variables as controls. Dependent variables were standardized. Standard errors are reported in parentheses.

	*dependent variable:*		
	attitude	incentivized estimate	incentivized estimate difference
Republican	−0.304** (0.103)	0.078 (0.103)	−0.337** (0.103)
crime questionnaire	−0.186* (0.078)	−0.061 (0.078)	−0.185* (0.078)
Republican × crime questionnaire	0.669*** (0.146)	0.213 (0.146)	0.641*** (0.146)
gender male	0.131 (0.068)	0.076 (0.068)	0.038 (0.068)
age	−0.002 (0.003)	−0.004 (0.003)	−0.002 (0.003)
income	−0.005 (0.011)	0.026* (0.011)	0.002 (0.011)
university education	0.045 (0.083)	0.068 (0.084)	0.056 (0.084)
(intercept)	0.100 (0.152)	−0.079 (0.153)	0.145 (0.153)
*N*	902	902	902
*R* ^2^	0.028	0.022	0.023
*F* statistic (d.f. = 7; 894)	3.669***	2.920**	3.054**

*p < 0.05; **p < 0.01; ***p < 0.001.

### 3.2. Pre-registred analysis

**Figure 1.**
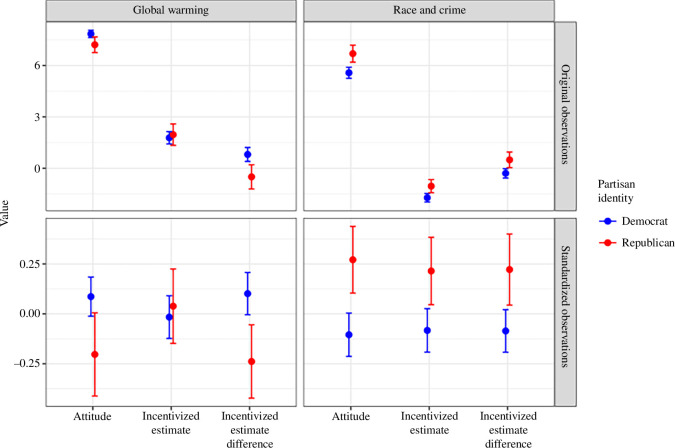
Distribution of the key variables measured in stages 1–3 by partisan identity and issue. Dots represent means and whiskers indicate 95% confidence intervals (CIs) of the mean. The top row shows the response on the scale at which the measures were taken, and the bottom row shows standardized values to enable comparison across questions and measures.

Figure 1 shows to what extent the answers for the three measurements depended on a participant’s partisan identity.^2^ The agreement with the statements on global warming and race and crime (labelled *attitude*) is separated along the expected partisan lines: Democrats agreed more with the statement that temperatures have risen in the United States during the last 100 years than Republicans, while Republicans agreed more than Democrats with the statement that there is a link between the racial composition of an area and the crime rate, which clearly supports H1. The acceptance of H3 is corroborated by the observation that Republicans and Democrats expect each other to have different incentivized estimates along partisan lines (*incentivized estimate difference* in figure 1).

The results are more ambivalent for the incentivized estimates, testing H2. No significant difference exists between partisan groups in the *Global warming* questionnaire. In other words, although Democrats and Republicans have different self-reported attitudes towards the reality of global warming, their incentivized estimates of temperature increases in selected US states do not differ. Conversely, differences between partisan groups were consistent for all three measures for the issue *Race and crime*: Republicans and Democrats have differing attitudes, give different incentivized estimates of real-world data and also expect each other to make different estimates. Regarding the incentivized estimate difference, we see that Democrats and Republicans correctly estimated each other’s average differences in the Race and crime questionnaire but clearly overestimated differences in the Global warming questionnaire. While H1 and H3 are, therefore, fully supported by the data, the effect of partisan identity on the incentivized estimates actually seems to depend on the issue at hand. H2 is therefore only partially supported.

Corroborating the insight gained by figure 1 and in accordance with the pre-registered analysis plan, table 2 formally tests the significance of the (in)consistencies between measures. Each of the three hypotheses was individually tested in one of the models. All models supported our hypotheses, with an interaction term that is positive and highly significant for both *attitude* and *incentivized estimate difference* but not significant for *estimate difference*.

### 3.3. Exploratory analysis

**Figure 2.**
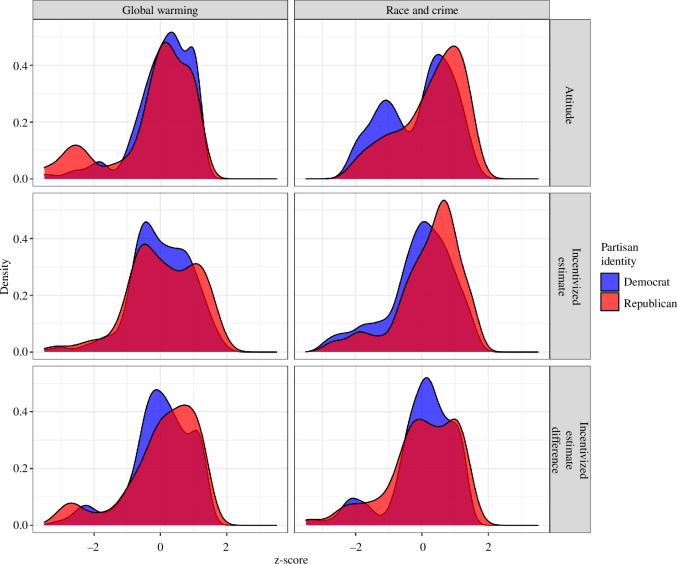
Distribution of the three key variables (after standardization) by questionnaire and partisan group.

To unpack heterogeneity within partisan groups, figure 2 shows the distribution of the three measures within groups. With respect to the modal tendencies within the distributions, negligible differences emerge between Republicans and Democrats. Moreover, these distributions exhibit significant overlap. Contrarily, when examining the distributions of self-reported attitudes from stage 1, a discernible subset of participants with extreme views becomes evident. This phenomenon is conspicuously absent in the incentivized estimates from stage 2 and manifests only modestly in the incentivized estimate differences from stage 3. Electronic supplementary material, table S4, reports formal analyses of multi-modality for all distributions shown in figure 2 and confirms that bi-modality is only present for the *attitude* measure.

In the context of Global warming, an extreme attitude group constitutes approximately 15% of Republicans, who vehemently oppose the assertion that temperatures have increased over the past century. For Race and crime, a similarly extreme group includes approximately 30% of the Democrats, strongly rejecting the claim that there are links between the distribution of racial groups and the crime rate in a given area. Similar groups of extreme participants cannot be found for the other two measures. A key result hence is that subgroups with extreme self-reported beliefs can be found in both partisan groups, but these stated beliefs are not reflected in the incentivized estimates of real-world data and in the expectations of what others believe. Consequently, our data indicate that self-reported beliefs possess scant, if any, predictive validity when it comes to individuals’ belief in actual evidence.

**Figure 3.**
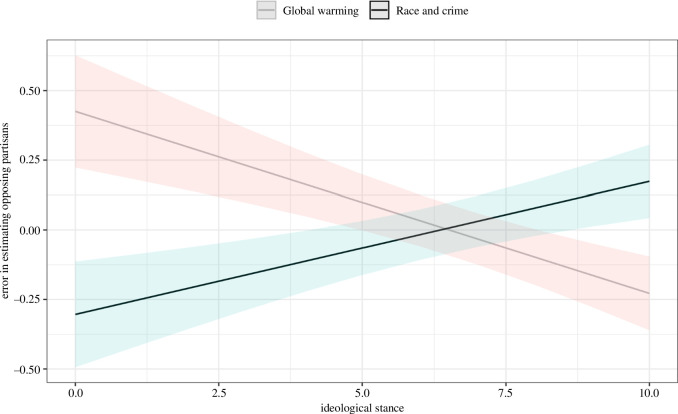
Marginal effect of ideological stance on the error of predicting estimates of opposing partisans. Shaded areas show 95% CIs. More detail on the regression model is in the electronic supplementary material.

Regarding our third measure, where participants had to predict the data estimate of a member of the opposing partisan group, we see that moderate individuals who do not clearly prefer one party are better at predicting others. Figure 3 shows the marginal effects of self-reported ideological stance (on a left–right spectrum) on the error of predicting others. Moderate participants with ideological stances close to 5 seem to have more realistic expectations of polarization, that is, made smaller errors when estimating other guesses and appear to be less vulnerable to ‘false polarization’.

Complementing our analysis, we tested for correlations among participants regarding the three measures. However, table 3 shows that correlations are low across most measures. The only substantial correlation can be found between the two incentivized measures (i.e. estimate and estimate difference), further strengthening our finding that attitudes are poor predictors of incentivized estimates.

**Table 3.** Per pair of measures, correlations of z-scores within partisan groups. All *p*-values are Bonferroni-corrected for multiple testing.

correlation	partisan preference	global warming	race and crime
attitude: estimate	Democrats	0.12	0.26***
	Republicans	0.24	0.23
attitude: estimate difference	Democrats	0.04	0.09
	Republicans	0.05	0.07
estimate: estimate difference	Democrats	0.40***	0.52***
	Republicans	0.49***	0.58***

*p < 0.05; **p < 0.01; ***p < 0.001.

## 4. Discussion

This study reveals that despite apparent polarization in expressed attitudes and mutual perceptions, partisan groups may be less divided in their actual beliefs about real-world data. Specifically, in the case of global warming, incentivized estimates from Republicans and Democrats align closely, indicating a reduced level of actual polarization compared with their stated attitudes. In contrast, for the race and crime issue attitudes, incentivized estimates, and expectations consistently align, reflecting a more straightforward relationship between expressed views and underlying beliefs. Moreover, and in line with the concept of false polarization, we find that partisans’ expectations about the opposing group’s adherence to data tend to exaggerate the actual differences. This nuanced portrayal of political polarization complicates the direct linkage between partisan attitudes and empirical beliefs, highlighting the complex dynamics at play in the context of polarizing issues.

One plausible explanation for this discrepancy is that Republicans, despite publicly denying the existence of global warming, may privately acknowledge its validity. This could account for their statistically comparable estimates to those generated by Democrats (average absolute error: Democrats = 2.88 ± 0.13°F, Republicans = 3.25 ± 0.22°F; Wilcoxon rank sum test, *W* = 19 340, *p* = 0.118 one-sided). Additionally, inter-party trust disparities in the data for this topic are statistically insignificant (see electronic supplementary material, figure S3). This observation undermines the notion that Republicans may have adjusted their estimates in the Global warming questionnaire to align with data they potentially consider faked or biased.

Conversely, Democrats appear to be confident that empirical evidence aligns with their views on the lack of a statistical association between race and crime. As a result, their incentivized estimates are statistically less accurate than those offered by Republicans (average absolute error: Republicans = 168 ± 15%, Democrats = 212 ± 11%; Wilcoxon rank sum test, *W* = 24 274, *p* = 0.004 one-sided). Importantly, this pattern persists even after accounting for covariates, such as age, gender, education, income and trust in data sources, as indicated in electronic supplementary material, table S3.

In general, we assume that participants carefully read and processed both the incentivized and unincentivized questions and that any inconsistencies between the answers are, therefore, the results of a deliberative choice. Nonetheless, existing literature indicates that incentives tend to promote more thoughtful decision-making [30]. In other words, it might also be true that participants did not change their beliefs or strategically choose to answer differently because of the incentives, but that they reevaluated what was asked of them because of the incentives. This distinction about the cognitive mechanisms at play is similar to the logic of System-1 and System-2 thinking [31] and more nuanced experimental designs are needed to identify the exact process at play. Furthermore, generating specific point estimates could be more cognitively taxing than merely indicating agreement with a statement. This complexity introduces a potential confounding variable when juxtaposing attitudes with incentivized measures. In this sense, incentives are inevitably linked with cognitive effort, while partisan-motivated reasoning has been shown to negatively correlate with cognitive effort [32]. Furthermore, a consistency bias [26], potentially introduced by the fact that participants were asked first about their attitudes before estimating the data, may render our study a conservative test for inconsistencies between attitudes and data estimates. At the same time, it is unclear why this bias should only operate for Democrats in the *Race and crime* questionnaire and not for Republicans in the *Global warming* one.

Determining the extent to which our results generalize to other polarized topics falls outside the purview of this study, although it is likely that issues exist where Republicans really expect real-world data to be consistent with their (factually wrong) beliefs. Nonetheless, one overarching conclusion with salient methodological implications is this: absent incentivized measures, it remains indeterminable whether self-reported attitudes on politicized issues merely reflect partisan stereotypes or signify deeply ingrained beliefs capable of influencing actual behaviour, especially when tangible consequences are involved. This underlines the imperative for future research to systematically examine the attitude–behaviour gap across a diverse range of politically polarized issues.

One plausible criticism of our findings could focus on the relatively low monetary incentives in our study—up to $4—which may not be sufficient to persuade individuals to abandon entrenched beliefs for more objective data. This argument yields two notable corollaries. First, Democrats may display a higher propensity to forgo monetary rewards to maintain consistent answers in the race and crime scenario compared to Republicans in the climate change context. Second, escalated incentives could result in more uniform responses across both the ‘global warming’ and ‘race and crime’ conditions. To validate these corollaries, subsequent experiments could employ an expanded range of treatment groups with varying incentive levels. Nevertheless, systematic reviews suggest that the effect of the incentive level on actual behaviour is surprisingly modest in several economic games, often lower than that of variables such as age or gender [33–35], which may cast doubt on the idea that higher incentives would have qualitatively changed our results.

Notably, irrespective of their own estimates, participants from both political affiliations anticipate that members of the opposing party will act in congruence with their publicly declared attitudes. Polarization and its effects hence seem to be driven more by publicly expressed attitudes and expectations towards others than by actual behavioural differences. These findings align closely with academic discourses on ‘perceived’ versus ‘false’ polarization [21,36]. To the best of our knowledge, our design presents the first incentivized measure of perceived polarization, showing that actual polarization is less pronounced when using incentivized measures, but that expectations about others’ polarization still prevail despite the provision of incentives. The results bear consequential implications for initiatives aimed at depolarizing discourse to facilitate the adoption of more collaborative and evidence-based policies. Reporting estimates of polarization based on economically incentivized tasks instead of simple attitude statements may actually help to mitigate the vicious circle linking (biased) views of the opposite party to more extreme positions and, in the end, to more polarization [37].

More generally, our results suggest that a depolarization of the debate—for example, through participation and more careful communication about the potential effects of climate change and the measures for climate mitigation and adaptation—may have a stronger effect on building consensus for the needed policy changes than standard information and education initiatives [38–40]. This assumes heightened importance in contexts like our ‘race and crime’ questionnaire, where numerically minor but ideologically extreme partisan groups disproportionately exacerbate polarization.

A similar situation also occurs in other domains of social life—for example, the COVID pandemic [11]—where people in different groups are expected to publicly support and enforce their partisan positions, even when privately holding concerns about them [8]. In these cases, a strong social desirability bias deeply affects questionnaire-based measures of attitudes, even in conditions where participants are guaranteed to be anonymous. Consequently, conventional awareness-raising efforts appear ill-fated in contexts saturated with polarization [12,41,42].

The divergent patterns observed between expressed unincentivized attitudes and incentivized estimates for the two issues imply that relying solely on attitudinal surveys risks insufficient data in polarized scenarios. Such an approach could misguide policymakers into formulating ineffective strategies to address pressing issues [25]. Incorporating incentivized metrics may offer a more robust foundation for the formulation of efficacious evidence-based policies, particularly in fields like environmental management, crime prevention and public health [43–47].

## Data Availability

Data and methods are available at the Open Science Foundation repository using the following link [48]. Electronic supplementary material is available online [49].

## Footnotes

https://osf.io/kcjys.

All scripts and data needed to replicate the analysis can be found at  https://osf.io/gzj7t/.
